# Inkjet Printing of High Performance Transistors with Micron Order Chemically Set Gaps

**DOI:** 10.1038/s41598-017-01391-2

**Published:** 2017-04-26

**Authors:** Peter Mack Grubb, Harish Subbaraman, Saungeun Park, Deji Akinwande, Ray T. Chen

**Affiliations:** 1 The University of Texas at Austin, Austin, TX 78712 USA; 2 Boise State University, Boise, 83725 ID USA

## Abstract

This paper reports a 100% inkjet printed transistor with a short channel of approximately 1 µm with an operating speed up to 18.21 GHz. Printed electronics are a burgeoning area in electronics development, but are often stymied by the large minimum feature size. To combat this, techniques were developed to allow for the printings of much shorter transistor channels. The small gap size is achieved through the use of silver inks with different chemical properties to prevent mixing. The combination of the short channel and semiconducting carbon nanotubes (CNT) allows for an exceptional experimentally measured on/off ratio of 10^6^. This all inkjet printed transistor allows for the fabrication of devices using roll-to-roll methodologies with no additional overhead compared to current state of the art production methods.

## Introduction

In recent years, printed electronics has quickly emerged as an area of great interest in manufacturing. Based on its compatibility with flexible substrates and high production rates promised via the use of roll-to-roll (R2R) methodologies^1^, printed electronics has the potential to open doors to new types of devices and development paradigms.

Despite the great promise of this field, many of the existing technologies have significant limitations. One of the more difficult ones is the feature size, which is currently limited to ~30 microns using traditional inkjet methods^2^. Several different methodologies have been developed for working around these limitations, such as imprinting^3^ or flexographic printing^4^. While these methodologies allow for micron order feature sizes^5^, they require lithographically defined patterns/masks, minimizing the unique flexibility that inkjet printing allows.

For many applications, a feature size of ~30 microns is sufficient to have a major impact. However, for transistors, this feature size leaves much to be desired. For example, a 10 micron channel length places the channel length of a printed transistor on par with silicon microelectronic devices made in the early 1970’s^6^, severely limiting their utility in applications requiring high frequency operation. For printed transistors to be viable in most modern applications, a much shorter channel is required.

For printed technology to satisfy the current day requirements and become sustainable in the future, channel lengths under 1 μm are required. Several different methodologies have been developed for working around the fundamental printed channel length limitations, such as imprinting^7, 8^ and flexographic printing^4^. While these methodologies allow for micron order feature sizes^4^, they still require lithographically defined patterns/masks, minimizing the unique flexibility and low cost options that printing allows. Recently, a print-and-drag (PND) method was demonstrated to provide 2–13 μm channels^9^, and another process relying on mechanically splitting a printing metal pad by dragging a probe tip across to produce channels in the range of 200 nm–600 nm was demonstrated^9^, however, these methods are not scalable or compatible with additive manufacturing processes. In Zhao *et al*. and Sele *et al*.^10, 11^, 200–400 nm printed gaps using a self-aligned printing (SAP) process was demonstrated, in which, after printing one silver ink, its surface properties were modified to make it repel the next silver ink printed directly on top or next to it. While ultra-short gaps were achieved, the yield was poor. Yield was improved to >94% by Caironi *et al*.^12^ through printing single droplets next to a bar. This high yield was possible by letting the inks dry for 340 s to complete the process of dewetting before curing them. This wait time is detrimental in a high-rate manufacturing process. Moreover, single drop printing and surface modification increases the extra steps involved and makes the process complicated, since additional contact pads need to be precisely aligned and printed to interface with the drop pattern for transistor development.

Overcoming these limitations, in this paper, we demonstrate 100% inkjet printed transistors with channel lengths in the range of 300 nm to 2 μm. This short channel was enabled by printing silver nanoparticle ink solutions with differing chemical compositions (repulsive) next to each other for the source and drain. This gapping process (further detailed in the following sections) is repeatable since it relies on chemically opposing forces to define the gap, and eliminates the need for (a) additional surface modification steps after printing, (b) controlling the drop volume to a single droplet, and (c) any wait time before curing. Carbon nanotube (CNT) transistor devices were developed using these sub-micron gapped channels, and their DC and AC performance was tested. These devices exhibited high On/Off ratio of 10^6^ with a high operating frequency, ft of 18.21 GHz, which are one of the best results reported for printed transistor structures, hence opening up tremendous opportunities for low-cost development of printed integrated electronic systems. A total of 216 devices were tested with a yield of greater than 95%, thus demonstrating the true scalability of the process for achieving integrated systems.

## Fabrication Technique

The entire fabrication process was completed using a Fujifilm Dimatix DMP-2831^13^ printer for deposition. Individual printed material layers were thermally cured in an oven.

### Device Structure

**Figure 1:**
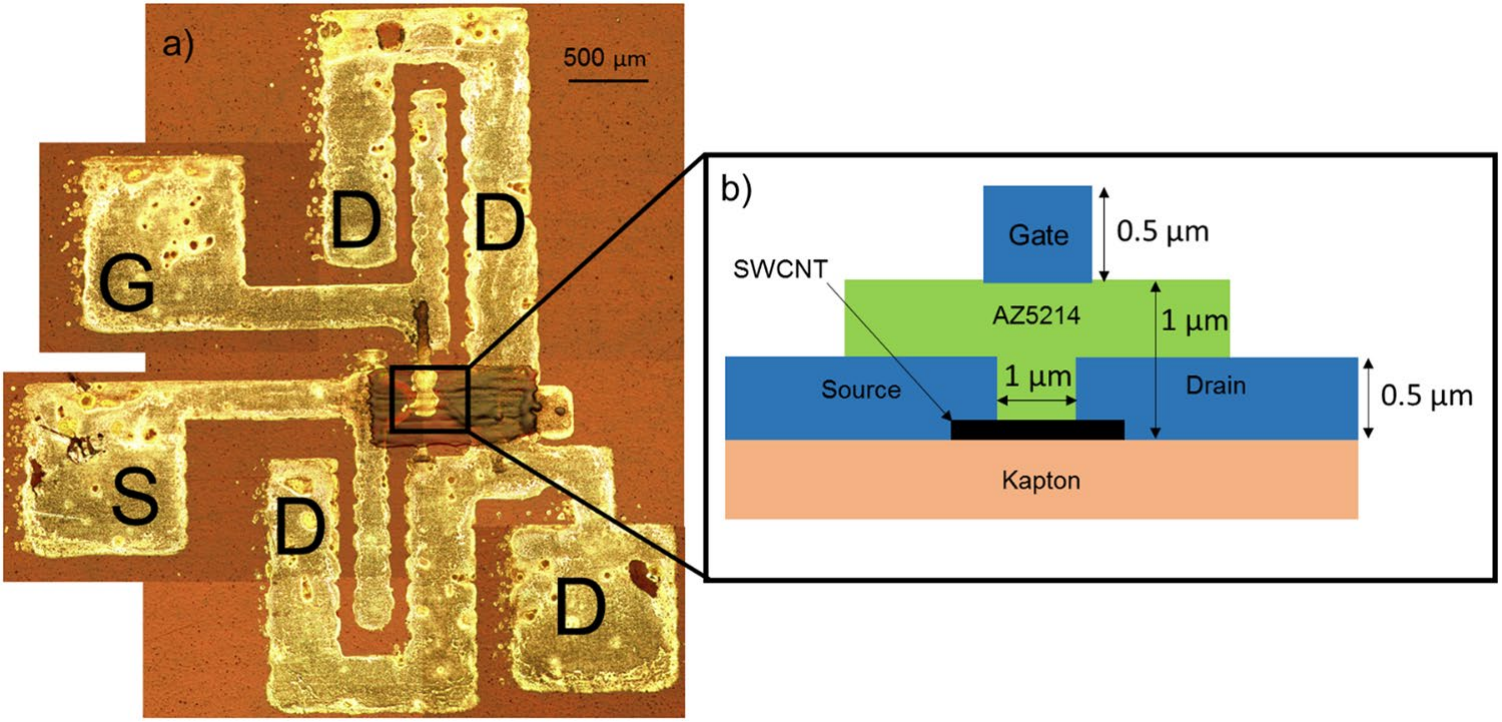
(**a**) A composite optical microscope image of the final completed device used for high frequency testing. G is the gate electrode, S is the source electrode, while the D’s are the common drain electrode. The structure is designed to allow RF testing with 500 micron pitch GSG probes. (**b**) A cross section of the top gate transistor design. Source, drain, and gate were all printed using nanoparticle silver solution.

The general device structure is shown in Fig. 1, with 1a showing the device layout and 1b showing the cross section of the transistor. A top gate structure was used in order to protect the SWCNT thin film and provide repeatable printability^14^. Without a protective top layer, the SWCNT thin film can dissolve on contact with environmental contaminants and liquids.

The substrate used was 500HN Kapton. This substrate provides a very high temperature tolerance ranging from −269 °C to 400 °C^15^, allowing for a wide range of compatible inks, while retaining high flexibility. Additionally, these properties make it an ideal substrate for use in aerospace and military applications.

First, SWCNT thin film structures were formed on the substrate using the techniques outlined in subsequent sections. Once the thin film was formed, the source and drain were printed on top, with one terminal using silver nanoparticle ink from Novacentrix Inc (JS-B40G) and the other using non-aqueous silver nanoparticle ink from UTDots Inc (UTDAg40IJ). The opposite polarities of inks cause the generation of a narrow, non-diminishing gap with a finite length. This process is discussed later in this paper. Silver was printed on top of the SWCNT thin film in order to maximize the contact surface area between the SWCNT network and the silver transmission lines. The source and drain were then cured at 200 degrees Celsius. Once this step was completed, a dielectric material (AZ5214 photoresist from MicroChemicals) was printed over the source, drain, and gap^16^. Finally, a gate was printed on top of the structure using the JS-B40G silver nanoparticle ink from Novacentrix.

The capacitance per unit area for the dielectric material was experimentally determined. Printing usually forms layers of very consistent heights, due to its high degree of control over the quantity of ink deposited in each layer. To find the value for C_diel_, a simple 100 μm × 100 μm silver patch was printed on top of a 1-micron thick layer of AZ5214. The capacitance across this structure was then measured allowing us to calculate C_diel_ to be ~9.3 nF/m^2^. The relatively low capacitance is achieved due to the relatively thick gate dielectric and lower dielectric constant of AZ5214.

### SWCNT Thin Film Printing

In the device outlined above the semiconductor consists of a network of single walled carbon nanotubes. Recent efforts for printed transistors have focused on the usage of semiconducting CNTs due to their exceptionally high mobilities^17, 18^. While other printable semiconductors have been used, none have shown the potential for the multiple GHz performance that CNTs have. While other bioFET devices have shown outstanding properties in terms of subthreshold swing and low gate voltage^19^, these devices have so far been unable to break into the GHz switching realm, whereas CNT devices have.

Typically, clean room processed SWCNT based transistors use some form of aligned SWCNTs^20^. This provides optimal performance while using the fewest number of CNTs. However, in recent years, the cost of CNTs has dropped to a point that using the minimal number is not necessarily a major concern from a cost standpoint^21^. While aligned thin films do provide significantly better performance^22, 23^, unaligned thin films are much easier to produce. Thus, efforts to ink jet print CNTs have largely focused on unaligned CNT networks^14, 18, 24^.

**Figure 2:**
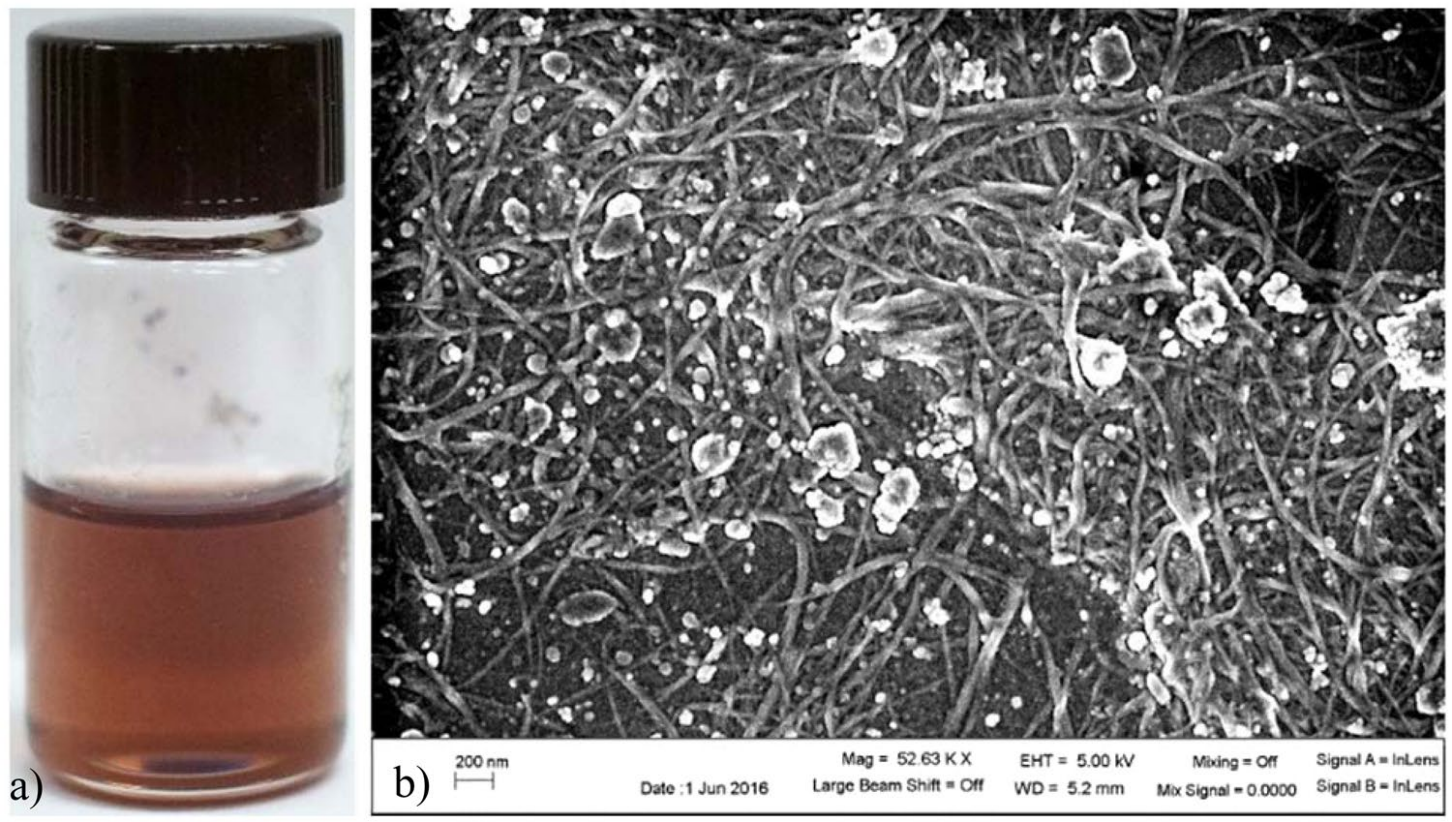
(**a**) A vial of SWCNT ink with a 20% CNT concentration by weight. (**b**) SEM image of a CNT film post-annealing. While there is residual from the solvent, conductivity is not impacted.

Past efforts to print these CNTs have largely been done using aerosol printer. This process has met with solid results^18, 25, 26^, but is not as flexible in terms of applications as ink deposition. Previously, our group formulated a mixture of CHP and SWCNTs which allowed the SWCNTs to be printed using a deposition printer^17^. CHP is ideal from a printing aspect for this application, as it has both a relatively low boiling point of 154 °C that is stable at room temperature, and a viscosity well suited to use in the Dimatix printing system^24^. However, while the CHP provides good print performance, subsequent layers must deal with the extreme hydrophobicity of the CNTs themselves. This means that the first layer of solution printed must be the only layer, as the CHP will be pushed away from the print site by the CNTs. To avoid this, a proprietary non-aqueous solution was developed with properties similar to the CHP. CNTs were dispersed via sonication in this proprietary solution at a 20% concentration by weight, leading to a solution like the one shown in Fig. 2a. This allowed for CNT printing without dealing with the aqueous/non-aqueous interactions of the CHP and CNTs.

Once the solution is deposited on the substrate, the proprietary solution is annealed away via thermal annealing. Figure 2b shows an SEM image of a multilayer CNT thin film after the annealing step. The final resistance of the CNT thin film was 200 kΩ, which was in line with past papers using aerosol printing^18^.

### Chemical Gapping Process

One of the primary challenges in fabricating a transistor via any printing methodology is the achievable minimum channel length. Traditionally, inkjet printed electronics achieves this gap between the source and drain by merely not printing a space in a transmission line. In theory this yields a gap where the width is limited by the resolution of the printer. Given that a printer like the Dimatix has a resolution of 10–30 µm depending on drop volume^13^, this provides a small gap, but by no means a short channel compared to photolithographic methods in a CMOS foundry^4, 5^.

In practice, this gap often has to be much larger. Not only does print position have fairly significant error bars, but this issue is further exacerbated by affinity of inks employed. Thus, if the source and drain are printed using the same ink, and the chemical force is greater than the surface energy of the substrate, the ink will pull across the source and drain, causing closure of the gap and creating a short circuit.

The concept of using surface effects to control gap size is not inherently new^11, 12, 27^. However, past efforts have focused on the usage of some sort of self-aligning monolayer. This potentially places major restrictions on both materials and substrates that could be used. Additionally, the technique has not been ported to a roll to roll technique, limiting its potential for mass production. In order to combat these disadvantages, the technique outlined below focuses instead on creating the chemical effect with multiple wet layers. This allows for the selection of any two inks with opposite chemical properties. For the specific device produced here, conductive silver inks with opposing chemistries were selected due to their easy commercial availability. Both inks form silver thin films when cured. However, one is a hydrophobic, non-aqueous hydrocarbon silver nano-particle solution while the other is a hydrophilic, aqueous silver nanoparticle solution. What this means is that the two inks cannot mix, similar to the way water and oil will separate when poured in the same glass.

**Figure 3:**
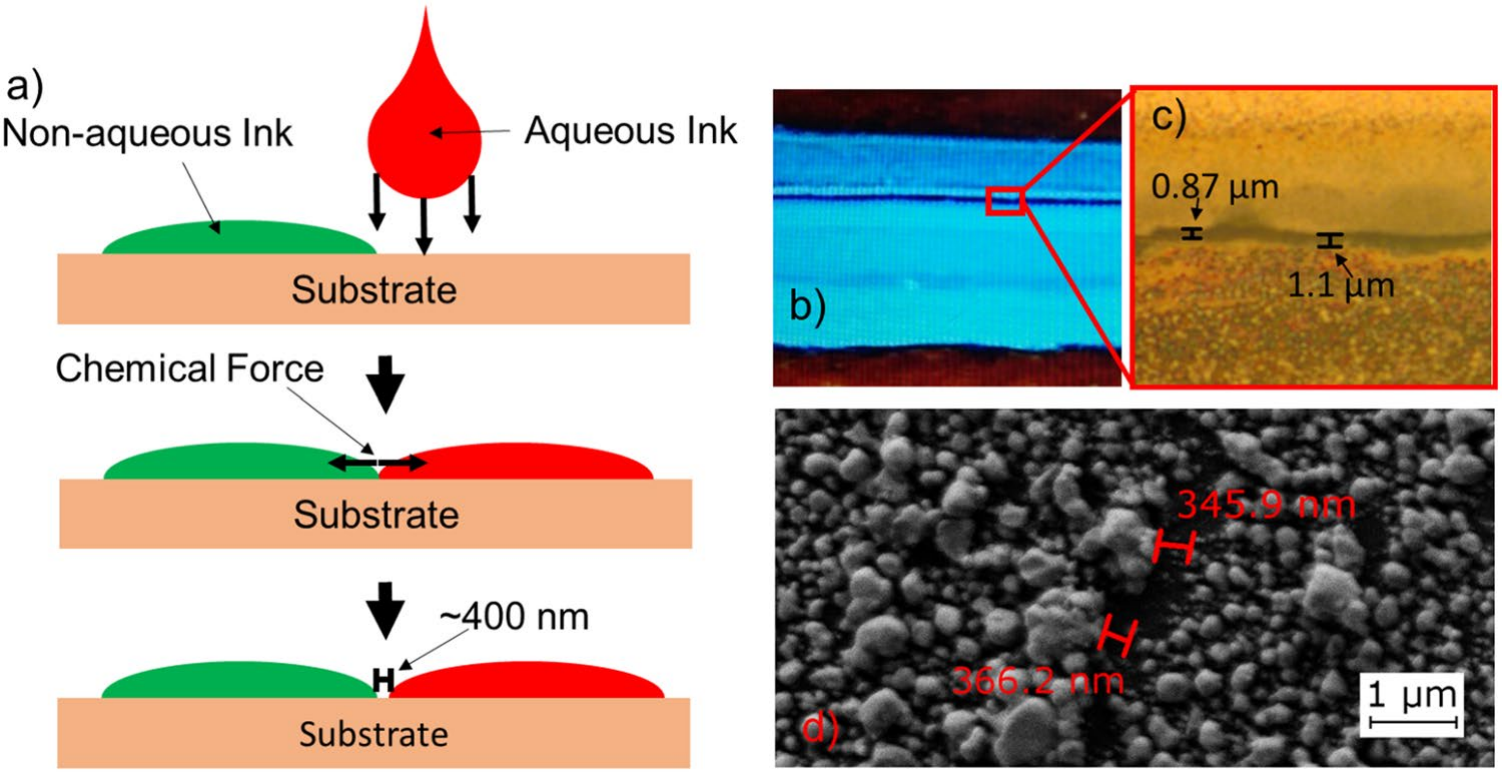
(**a**) Illustration of the chemical forces between the inks. A small gap can be accomplished with any ink pairing with opposing chemistries, not just silver nanoparticle solutions. (**b**) Two parallel lines forming a chemical gap. The lines are electrically isolated. (**c**) A zoomed in image of an average chemical gap. Results like this were observed across multiple devices. (**d**) SEM photograph taken at 16880× magnification of the smallest observed electrically isolated gap.

By printing the two inks in a single layer side by side, the chemical force would produce the gap as shown in Fig. 3a. In the printing process, the two ink sections were printed right next to each other with no gap from the printing process. However, the chemical forces between the two inks introduce a micron to sub-micron order gap.

One major advantage of this method over previously listed short channel methods is its flexibility and tunability. In our experiments, Kapton was used with two off-the-shelf inks. However, by using custom formulated silver inks, various solvent combinations could be used to introduce gaps with a specific length. Gap size would theoretically be controlled by the net polarity difference between the chosen solvents. This also allows the technique to be adapted to other substrates that have surface coatings that might affect the polarity of the substrate, and thus the interactions of the inks with it. As far as substrate limitations go, the primary issue would relate to surface roughness. At a certain surface roughness the ability of the inks to move apart might become impeded. However, this effect was not observed on Kapton, Glass or PET, which covers three of the most common substrates used in printed electronics.

Additionally, unlike most other short channel methods that have been developed for these types of devices, this technique is fully roll to roll compatible, and relies on the usage of inkjet deposition as opposed to gravure^28^ or offset methodologies^29^. Inkjet technology does not require the use of any kind of stencil or mask to produce devices, allowing designs to be changed at essentially no cost. This is in essence one of the core promises of inkjet deposition technology, which this technique does not interfere with in any way.

Additionally, this method has the added benefit of being highly reliable. Rather than relying on a mechanical process, this methodology relies on a chemical process which should be consistent as long as the formulation of the inks does not change. Thus, it is possible to print this gap much smaller without having conduction between the two sides of the gap while still maintaining print reliability.

## Results

Results from the fabrication can be split into two groups. The first shows results from the fabrication process itself, while the second focuses on the performance of the fabricated device.

### Average Gap Sizing

In order to characterize the performance of the gapping technique outlined previously, a series of 50 gaps was fabricated using the Dimatix printer in conjunction with the two different inks. Each gap was 1 mm long, with a single layer of ink 150–300 microns wide on each side, as shown in Fig. 3b. The resulting gaps were imaged using an optical microscope and a Zeiss Scanning Electron Microscope to find accurate measurements for each gap. Of the 50 gaps fabricated in this example only a single device was shorted. To further characterize the yield rate, during one transistor fabrication run, 216 gaps were fabricated, of which 10 were shorted. Based on these experiments, this particular combination of inks and substrate has a yield rate greater than 95%. However, it is worth noting that with custom ink tuning without proprietary solvents, a greater yield rate could be achieved.

Across all the optical measurements, a range of 0.5–2 micrometers was observed in gap size, with an average gap size of 1.1 microns and a standard deviation of 0.437 microns. Figure 3c shows an optical image of one of these gaps with observed measurements. It is worth noting that the Dimatix printer did have some alignment issues which contributed to the larger gap sizes when printing many simultaneous gaps for developing this average. With a more precise multi-layer printer with better interlayer alignment, more consistent gap sizing could be achieved.

In addition to the repeatability study, a separate set of fabrication was done to determine the minimum gap length that could be achieved using this particular combination of inks. As shown in Fig. 3d, gap sizes as small as 300 nm were observed using the Zeiss SEM. This was verified via capacitance measurements to ensure that the conduction was occurring all the way out to the edge of the silver deposition.

### Transistor Performance

**Figure 4:**
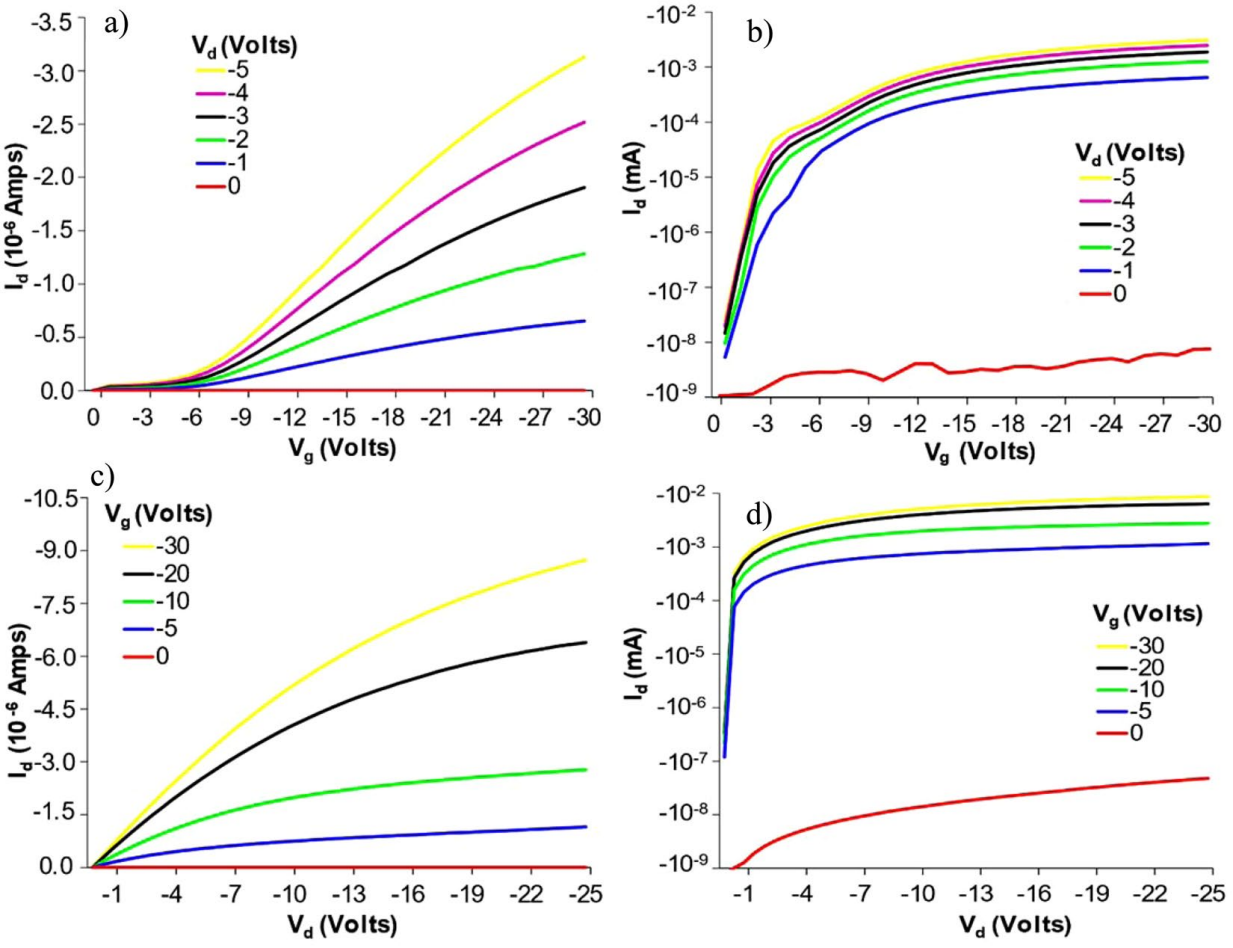
Id vs Vg plotted on a linear (**a**) and logarithmic (**b**) scale. Id vs Vd plot also plotted on a linear (**c**) and logarithmic (**d**) scale. All plots demonstrate the 10^6^ on/off ratio.

Two different tests were preformed on the transistor to determine its capabilities. The first was a simple DC analysis which was completed using an Agilent B1500A transistor analyzer. This analyzer was used to perform both a Vgs sweep and a Vds sweep in order to obtain a Id vs Vg and Id vs Vd plots. The resulting plots are shown in Fig. 4a,b and c,d, respectively. Of particular interest is the on-off ratio, which was found to be on the order of 10^6^. This is compared to previous efforts using unaligned printed SWCNT thin films, which achieve 10^2^ ^5, 18, 25^.

Based on the transfer characteristics shown above, the threshold voltage and mobility were calculated. By examining Fig. 4b, the threshold voltage is found to vary somewhat with V_d_ as is commong for most FET devices. At V_d_ = −1 V, the threshold voltage is approximately −7 volts, varying up to −11 volts at V_d_ = −5 V. These relatively high threshold voltages are a product of the acrylate dielectric used to produce these devices. Using an ion gel^26^ or hybrid^30^ dielectric would potentially decrease this number considerably. Mobility is found using the peak transconductance ${G}_{m}=\mu {C}_{gc}\frac{W}{L}{V}_{DS}$, where *C*_*gc*_ is the gate capacitance. As stated previously, the capacitance of the gate dielectric is 9.3 nF/m^2^. Using an optical microscope, the channel area was found to be approximately 10 square microns with dimensions of L = 0.8 microns and W = 12.5 microns, yielding a C_gc_ of 0.093 pF. Using the peak transconductance from V_DS_ = −2 V in the linear reagion, it was found that the effective mobility of the device was aproximately 6 cm^2^/(V s), well in line with other CNT devices with similar on-off ratios^31^.

The second test was to determine the maximum switching frequency of the device. Given that past efforts by the group yielded devices operating up to 5 GHz^18^, new techniques were needed to determine the max frequency which should theoretically be higher due to the much shorter channel length. To measure a transistor’s maximum frequency via RF tools, the parameter of interest becomes H_21_, or the small signal gain of the transistor^32, 33^. When the small signal gain is 0, the transistor has reached maximum operating frequency. The H parameter may be derived using the S parameters measured by a network analyzer and the equation ${H}_{21}=-\frac{{S}_{21}}{(1-{S}_{11})(1+{S}_{22})+{S}_{12}{S}_{21}}$, where port 1 is the gate and port 2 is the source. This allows the use of RF equipment that will usually be capable of 5+ GHz measurements, rather than lower speed traditional transistor measurement equipment.

**Figure 5:**
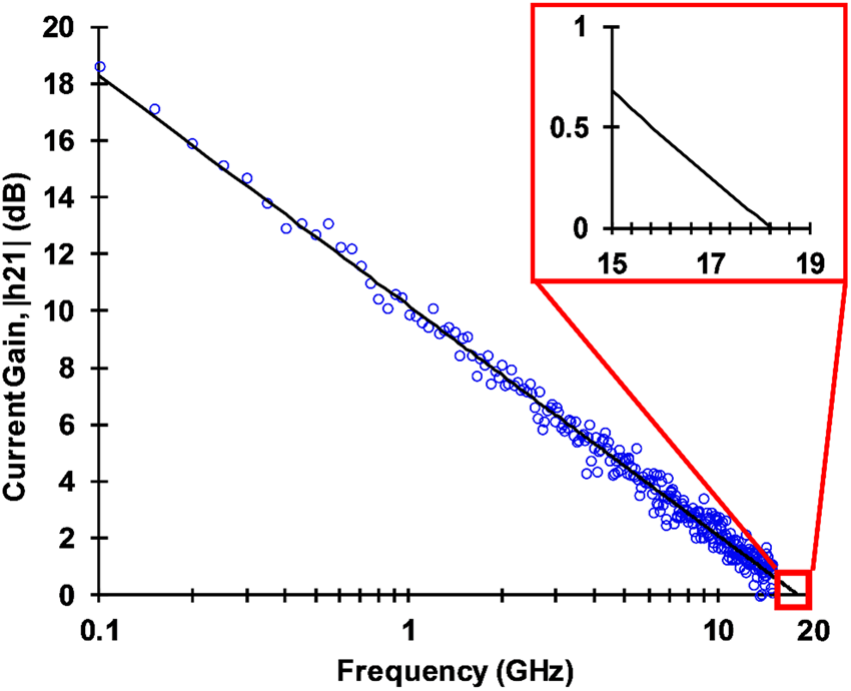
(**a**) De-embedded current gain with an intrinsic f_t_ of 18.21 GHz. (**b**) Detail showing point where H_21_ = 0.

The transistor pictured earlier in Fig. 2 was measured using this methodology, with the drain tied to the ground plane on a pair of GSG probes and the source and gate serving as the signal lines. The measured transit frequency for the extrinsic device as whole yielded the parameter f_t,ext_. Typically, f_t_ is significantly larger than f_t,ext_ due to parasitic capcitance and resistive effects. Thus, an experimental dembedded value which actually derives f_t_ is required. This is accomplished via standard two step dembedding to derive the performance curve for the actual device independent of these parasitic effects^34, 35^. This method essentiallty consists of measuring the extrinsic device response and then an open and short test structure on the same substrate. With this data, the intrinsic f_t_ is derived, yielding a de-embedded value for the transistor performance. The intrinsic data set is shown in Fig. 5. The extrapolated line showed that the transistor was capable of operating at up to 18.21 GHz (at the point when h_21_ = 0).

To ensure that experimental de-embedded values are reasonable, an analytical estimate needed to be made for the device. For intrinsic results, ${f}_{t}={g}_{m}/2\pi {C}_{gc}$ where g_m_ is intrinsic transconductance and C_gc_ is the gate capacitance. C_gc_ was calculated above to be 0.093 pF. The parameter g_m_ can be extracted from the extrinsic transconductance G_m_ (which is found using the DC curves for the device) via the equation ${g}_{m}=\frac{{G}_{m}}{1-{G}_{m}[{R}_{s}+({R}_{s}+{R}_{D})/({G}_{m}/{G}_{ds})]}\,$, where G_DS_ is the extrinsic device conductance, and R_s_ and R_d_ are the source and drain resistances respectively. Using this equation, g_m_ was found to be 13.3 mS at the drain bias point used for gathering the high frequency data. When this was subsituted to the intrinsic predictor, the analytical result of ~22.76 GHz was found. Compared to our actual measured result, these analytical results show that the device was operating as expected.

These results represent some of the best performance seen in any printed electronics device to date. Through the use of high purity CNTs and novel production methodologies, high frequency switching has been demonstrated this creates the possibility for inkjet printed amplifiers and other high frequency RF devices.

### Future Exploration

Several areas of this project look to need further research in order to fully develop. First and foremost relates to ink formulation. In theory, ink pairs could be “tuned” to find the ideal chemical force for minimal gap size. Currently, this is not possible given the proprietary nature of the inks involved. However, through collaboration with ink manufacturers such tuning could be achieved.

Additionally, further research is needed into finding the ideal way to print these inks. Layer count, temperature, annealing profile, and ambient humidity can all play into the inkjet printing results. It will be important to examine how this affects the chemical gapping.

Finally, this technique uses components which are all roll-to-roll compatible^1, 36^. This means that in theory these devices could be produced in large quantity at manufacturing level throughput. Yield rate and performance studies of the devices produced in such a system are a necessary next step in verifying this potential application.

## Conclusion

In conclusion, a thin film transistor with a high on/off ratio of 10^6^ was fabricated. This high on/off ratio was made possible by the use of a novel chemical gapping technique, which exploited chemical differences in silver ink formulations to create a gap in the range of 0.3–2 µm. This short channel device also exhibited exceptional switching capabilities, demonstrating high frequency operation up to 18.21 GHz. To the best of our knowledge, this represents one of the first printed devices which exhibits properties similar to CMOS fabricated devices.
